# Profiling strugglers in a graduate-entry medicine course at Nottingham: a retrospective case study

**DOI:** 10.1186/1472-6920-12-124

**Published:** 2012-12-18

**Authors:** Paul Garrud, Janet Yates

**Affiliations:** 1 School of Graduate Entry Medicine & Health, Royal Derby Hospital, Uttoxeter Road, Derby, DE22 3DT, UK; 2 Medical Education Unit, Medical School, Queen’s Medical Centre, Nottingham, NG7 2UH, UK

**Keywords:** Graduate-entry medicine struggler identification flags UK

## Abstract

**Background:**

10-15% of students struggle at some point in their medicine course. Risk factors include weaker academic qualifications, male gender, mental illness, UK ethnic minority status, and poor study skills. Recent research on an undergraduate medicine course provided a toolkit to aid early identification of students likely to struggle, who can be targeted by established support and study interventions. The present study sought to extend this work by investigating the number and characteristics of strugglers on a graduate-entry medicine (GEM) programme.

**Methods:**

A retrospective study of four GEM entry cohorts (2003–6) was carried out. All students who had demonstrated unsatisfactory progress or left prematurely were included. Any information about academic, administrative, personal, or social difficulties, were extracted from their course progress files into a customised database and examined.

**Results:**

362 students were admitted to the course, and 53 (14.6%) were identified for the study, of whom 15 (4.1%) did not complete the course. Students in the study group differed from the others in having a higher proportion of 2ii first degrees, and scoring less well on GAMSAT, an aptitude test used for admission. Within the study group, it proved possible to categorise students into the same groups previously reported (struggler throughout, pre-clinical struggler, clinical struggler, health-related struggler, borderline struggler) and to identify the majority using a number of flags for early difficulties. These flags included: missed attendance, unsatisfactory attitude or behaviour, health problems, social/family problems, failure to complete immunity status checks, and attendance at academic progress committee.

**Conclusions:**

Problems encountered in a graduate-entry medicine course were comparable to those reported in a corresponding undergraduate programme. A toolkit of academic and non-academic flags of difficulty can be used for early identification of many who will struggle, and could be used to target appropriate support and interventions.

## Background

A common observation is that around one in ten students at UK medical school struggle at some point in their course. This happens despite the considerable competition for places and selection primarily on the basis of high levels of achievement in prior education. Recent studies [1-5] have documented a number of risk factors and investigated characteristics of medical students who have encountered academic difficulty whilst undertaking 5-year undergraduate courses. Risk factors associated with difficulty include weaker entry qualifications, mental illness, male gender, UK minority ethnicity status, and poor study skills. Related work has also prompted considerable interest and enquiry into suitable and effective support and intervention [6-8]. Recently, Yates [1] published a toolkit to aid early identification of students who are likely to struggle, in which warning flags can be set for a variety of factors (e.g. exam failure, failure to attend Hep B screening). Nearly all this work has concerned medicine courses taken in the UK predominantly by students progressing directly from secondary education around the age of 18 years. Much less information is available concerning the graduate entry medicine programmes established in the UK over the last decade. Published evidence [9-11] suggests that higher proportions of graduates complete their medicine programmes, and that there may be fewer academic failures amongst these groups compared to groups of school leavers undertaking medicine. The present study reports a parallel study to Yates [1] that investigates the number and characteristics of strugglers on a graduate entry medicine programme.

The University of Nottingham has run a 5-year medicine programme since 1969. In 2003 it also started a 4-year graduate entry medicine (GEM) programme with a condensed largely pre-clinical phase (18 months c.f. 30 months in 5-yr programme). The first four entry cohorts (2003–6) were selected for this study. Ethical approval was granted by the University of Nottingham Research Ethics Committee, ref B/11/2009. The specific research questions were:

• What are the patterns of difficulty, attrition and course disruption amongst graduate entry medicine students?

• What health and social issues are associated with course disruption?

• How can one identify early those who struggle?

## Methods

The methods used were essentially those reported in Yates [1] and are not reproduced in detail here.

### Identification of target group

GEM students from the 2003–2006 entry cohorts who had demonstrated unsatisfactory progress at any stage or who had left prematurely were studied. Struggling GEM students were identified in several ways, viz: those seen at the Academic Progress Committee during the pre-clinical phase (first 18 months) were identified by the GEM Course Office. Any GEM students who had had difficulties during the clinical years were identified via notes from the Academic Progress Committee at Nottingham, notes made by the Clinical Sub-Deans, and any who had failed Finals. Archived files were also searched for those who had failed to graduate.

Additional file 1 provides a general description of the GEM course structure and selection criteria.

### Data extraction and analysis

Course progress files were hand-searched and all relevant data extracted into a customised Access database. Discrete yes/no categories of information were supplemented by free-text boxes. Pre-admission information included age, gender, domicile, declared disability, class of first degree, and performance on the two selection criteria – GAMSAT (unweighted mean score over 3 sections, weighted mean score – section 3 - reasoning in biological & physical sciences - double weighted) and interview. Summary variables plus free text were used to record course progress but actual examination marks were not used.

We categorised students as:

• ‘Struggler’ with multiple problems throughout the course

• ‘Preclinical’ - problems largely confined to the first 18 months

• ‘Clinical’ - problems largely confined to the later years

• ‘Health-related’ - problems largely related to ill health

• ‘Borderline performance’ - weak student, generally low marks throughout

• ‘No substantial problems’. Some students who were identified, for example, via APC attendance, had actually suffered only a minor or one-off drop in performance, and were subsequently eliminated from the database

• Left the course voluntarily

• Course terminated

Further variables were generated as required during the analysis, to create ‘flags’, and these are described in the Results section.

Data were first checked and cleaned, then analysed in Access or SPSS v17. Free text was printed out in report format so that it could be reviewed for themes such as poor attendance, adverse behaviour, health issues etc., and then additional ‘flags’ added to the database.

## Results

A total of 362 students entered the GEM programme over the 4 years 2003–6. In all, 53 (14.6%) were identified for this study. Table 1 provides a breakdown of the numbers in each category of ‘struggler’, and also includes comparative figures from the Nottingham undergraduate medicine course previously reported [1].

**Table 1:** Categories of student identified

	**GEM**	***UG***^***+***^
**Completed the course**	**n = 38**	***n = 87***
Struggler – problems in both pre-clinical & clinical parts of course	8	*25*
Pre-clinical problems predominated	3	*18*
Clinical course problems predominated	5	*8*
Problems largely health related	14	*17*
Borderline performance	8	*19*
**Did not complete the course**	**n = 15**	***n = 75***
Still on course, discarded from database	1	*2*
Left course voluntarily	8*	*59*
Course terminated	6	*14*

*2 students left the course in the initial weeks – no relevant data was identified.

+ figures in italics from Nottingham UG medicine course [1].

There was no significant difference in the proportion of students who struggled on the GEM compared to the UG course (p = 0.30). There was a significant difference in the proportions that completed or did not complete the course between the GEM and UG courses (Chi square = 5.31, df 1, p = 0.021): a higher proportion of GEM strugglers completing their course.

Three students were eliminated from the database: one who is still on an extended course, and two who left within the first few weeks of the course, leaving a total of 50 who constituted the study group; the remaining 309 students in these cohorts constituted the comparison group.

There were no differences (p > 0.10) between the study and comparison groups in terms of gender. However, there was a reliable difference in terms of classification of their first degrees: those with poorer initial degrees (2ii or 3rd class) were more likely to be in the study group (p = 0.017; OR = 2.03, 95% CI 1.29-3.57) than those with 1st or 2i degrees. A summary of demographic and qualifications characteristics is shown in Table 2.

**Table 2:** Comparison of student groups

**Descriptor**		**Students making normal progress (comparison group)**		**Early leavers and students failing to thrive (study group)**				**OR**
		**N = 309**	**%**	**N = 53**	**%**	***χ***^***2***^	**p**	**95% CI**
Sex	Female	128	41.4	23	43.4	0.072	NS	
	Male	181	58.6	30	56.6			
Degree Faculty	Biological or life science	127	41.2	21	39.6			
	Health professional qualification	17	5.5	3	5.7			
	Natural science, engineering, maths or IT	89	28.9	14	26.4			
	Humanities, law, social sciences or arts	76	24.7	15	28.3			
Degree Faculty group	Biological or life science	127	41.1	21	39.6	0.041	NS	
	All others	182	58.9	32	60.4			
Degree class *	1st (or GPA equivalent)	52	16.9	8	15.1			
	2.1	154	50.0	19	35.8			
	2.2	96	31.2	25	47.2			
	3rd	3	1.0	1	1.9			
	Other (Higher degree, Masters/Doctorate)	3	1.0	0	0			
Degree class group *	1st, 2.1 or higher degree	209	67.9	27	50.9	5.72	0.017	2.03 1.29 – 3.67
	2.2 or 3rd class	99	32.1	26	49.1			

*data not available for 1 non-study student.

**Figure 1:**
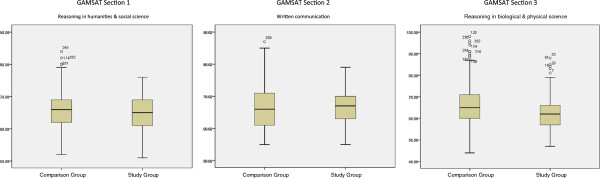
Boxplots of GAMSAT section scores.

The study group also differed significantly from the comparison group in terms of one of the two measures used to decide admission to the programme – their overall GAMSAT score. As the distribution of scores were significantly skewed (Kolmogorov-Smirnov tests, ps < 0.01), Mann–Whitney U tests were used: median GAMSAT overall score (unweighted mean) was non-significantly lower for the study than the comparison group (65.0 vs 66.1, z = −1.766, p = 0.077), and significantly lower for the weighted mean overall score (64.4 vs 66.1, z = −2.589, p = 0.010). Analysis of the separate section scores (GAMSAT comprises three separate tests) showed a non-significant trend toward lower scores on section 3 (reasoning in biological and physical sciences; means and SDs: Strugglers 63.28 ± 9.56, Comparison group 65.99 ± 9.64; p = 0.06), but no difference in scores on sections 1 or 2 (means and SDs: Strugglers 65.36 ± 5.17, 66.42 ± 5.93, Comparison group 65.64 ± 5.31, 66.40 ± 6.58; p = 0.72, 0.99 respectively). Figure 1 shows box plots of the GAMSAT scores for each group on each section. There was no reliable difference in terms of interview performance.

**Figure 2:**
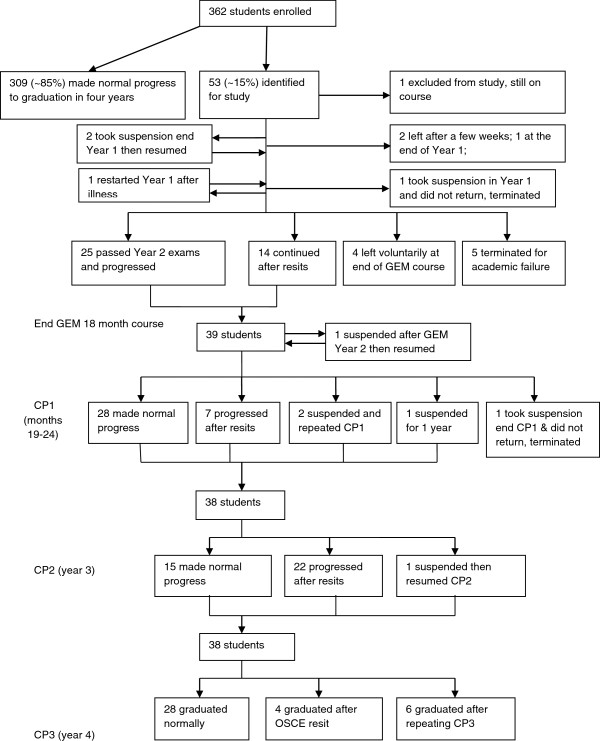
Flow chart of course progress.

Figure 2 shows the flow of students through the programme and the stage at which abnormal progress or attrition occurred for the study group. Of those who left, two left within the first few weeks, one took suspension in year 1 but did not return, four left voluntarily after their 2nd year (i.e. at the end of the pre-clinical phase), and five had their course terminated after academic failure. The remaining 38 all completed the programme, graduating after various difficulties or delays. Overall, the attrition rate was 15 out of 362 (4.1%), lower than reported for the 5-year undergraduate medicine programme at Nottingham (6.1%, [1]).

Yates [1] showed that presence of adverse health, social, or other circumstances early in the course were associated with poor progress and/or greater attrition. Similar flags, therefore, were created for the study group over the 18 months pre-clinical course, namely:

• Missed attendance noted

• Unsatisfactory attitude or behaviour noted

• Affected by health problems

• Affected by social or family problems

• Failed to complete Hepatitis B immunisation or to notify Occupational Health service of immune status

• Attendance at Academic Progress Committee

Table 3 shows the total number of flags combined for each individual in the study group. The numbers are small, but the pattern suggests that many of those destined to struggle or leave may be identified in this period: overall, 26/49 (53%) had two or more flags by this stage (end of first 18 months), and of those who left the course 9/11 (82%) had two or more flags. Amongst those who left, examination of the free text comments showed that many had health problems and these were nearly all psychological – some suffering recurrence of previous illness, others associated with bereavement or with living away from family or partners. Several also had financial difficulties. A small number also had notes of unsatisfactory attitude or behaviour. Some also failed to disclose adverse circumstances until their unsatisfactory progress meant their course of study was likely to be terminated.

**Table 3:** Flags in the first 18 months, pre-clinical course, by student category

**Number of flags for first 18 months (attitude, attendance, health, social, vaccs & APC)**	**Struggler**	**Preclinical problems**	**Clinical problems**	**Weak / Borderline**	**Health-related problems**	Left voluntarily *	**Course terminated**	**All students**
	**n = 8**	**n = 3**	**n = 5**	**n = 8**	**n = 14**	**n = 6**	**n = 5**	**n = 49**
0	0	1	3	3	2	2	0	11
1	2	0	1	3	6	0	0	12
2	3	1	1	1	3	1	1	12
3	1	1	0	1	3	1	1	7
4	1	0	0	0	0	0	2	4
5	1	0	0	0	0	1	1	2
6	0	0	0	0	0	1	0	1
Proportion with 2 or more flags	6/8	2/3	1/5	2/8	6/14	4/6	5/5	26/49

*Two further students left so early that there were no flags recorded, and a third had no course file available.

A number of the study group had new or continuing difficulties during the full-time clinical rotations (i.e. the last 30 months of the programme) and so the corresponding flags were added for this latter part of the programme, including separate flags for attendance at the Academic Progress Committee in each of the three clinical phases. Table 4 shows the total numbers of flags for the 38 students from the study group who eventually graduated. Overall, 22 of the 38 (58%) had 2 or more flags, and the highest proportions were in the health-related, and the strugglers group. Amongst those with health-related problems who eventually graduated, like those who left the course, mental health predominated, with anxiety, depression and chronic fatigue, some of which seemed likely related to recorded social and personal circumstances, including relationship breakdown, accommodation problems, social isolation, and worries about family members. This pattern is similar to that reported by Yates [1] for undergraduate medicine. Only one admitted to financial difficulty.

**Table 4:** Flags in the last 30 months, full-time clinical rotations, by student category

Number of flags for last 30 months (attitude, attendance, health, social, and APC*3)	**Struggler**	**Preclinical problems**	**Clinical problems**	**Weak / borderline**	**Health-related problems**	**All students**
	**n = 8**	**n = 3**	**n = 5**	**n = 8**	**n = 14**	**n = 38**
0	1	2	0	2	1	6
1	0	1	2	3	4	10
2	3	0	1	3	3	10
3	3	0	1	0	3	7
4	0	0	1	0	3	4
5	1	0	0	0	0	1
Proportion with 2 or more flags	7/8	0	3/5	3/8	8/11	22/38

One other instance of adverse behaviour was examined separately – failing to comply with requirements around immunity status (mostly Hepatitis B). In a medicine course this suggests unprofessional behaviour. The highest frequency of this adverse behaviour was in the strugglers group, where it was recorded for 4/8 (50%).

## Discussion

In this small study, a similar proportion of GEM students experienced difficulties as has been reported in the corresponding undergraduate programme at Nottingham [1]. Over half of those encountering difficulty, resulting in disrupted progress or departure from the programme, could be identified by a count of two or more flags – markers of difficulty - in the first 18 months. These flags characterised an even higher proportion of those who left prematurely. Similarly, over half of those remaining on course who continued to experience difficulty in the remaining 30 months before graduation, could also be identified by flags of difficulty in the full-time clinical rotations. It proved possible to categorise GEM students encountering difficulty in the same way as Yates [1]. One group – those with health-related difficulties – predominantly suffered from psychological illness. There was also some association between struggling and unprofessional behaviour, flagged as non-compliance around immunity status.

These results provide a little evidence about predictors, or risk factors, for difficulty on a graduate entry medicine programme. Overall, the pattern of difficulty and the associated markers are similar to those reported in studies of undergraduate medicine programmes in UK [1-5]). In the present study weaker prior academic attainment was associated with difficulty in the medicine course, as indicated by a poorer first degree and lower GAMSAT score – this last probably indicating an initial shortcoming in basic biological concepts and processes. Academically weaker students are known to be at greater risk of struggling in medicine [2,12,13], but this result may be a feature of the Nottingham GEM programme, that admits a proportion of applicants with lower 2nd class degrees, and not necessarily some other UK graduate entry programmes. GAMSAT is used in this programme, as others, partly as an indicator of applicants’ capability to tackle the medical sciences component of the course since many applicants do not have educational qualifications in science. Recent research has produced conflicting data about the predictive validity of GAMSAT [14-17]; the present results may indicate its value, and the section assessing reasoning in biological and physical science specifically, in identifying students without a science background who struggle with the early medicine curriculum.

One feature of interest is that all of those in the study group who completed the first 18 months, largely pre-clinical, part of the GEM course and continued into the full-time clinical rotations, subsequently graduated: all the attrition occurred in that first phase: this differs significantly from the pattern previously reported in the Nottingham undergraduate medicine programme [1], though it is closer to the pattern found in other research on the ‘failure to fail’ students during clinical placements [18]. Indeed, the overall attrition rate was lower than that in the Nottingham undergraduate medicine course, also reported by Manning & Garrud [9]. This raises the question of the curricular strategy employed in many of the UK fast-track, 4-year GEM programmes, that truncates the pre-clinical stage (from 30 to 18, or from 24 to 12 months) while maintaining the length of the clinical rotations. This clearly has resulted in an intensive initial phase of these medicine programmes and may be responsible for the pattern of attrition found in this study.

Since there was a substantial proportion of health-related difficulties, almost all comprising psychological illness, one question is whether risk of mental illness can be picked up as part of the selection process. In common with all UK medicine programmes, Nottingham commissions an independent occupational health assessment for each entering medical student: very rarely does this assessment indicate that a student is unfit to study or train, though recommendations for support are more common [19]. As pre-existing chronic health conditions may be construed as a disability under the UK Equality Act 2010 [20], a past history of depression or an eating disorder cannot lawfully be a criterion on which to refuse entry per se. However, many selection processes do attempt to assess resilience or stress management on the basis that training and working as a doctor involves high workload and emotional demands [21,22]; however, the present study did not find any evidence linking performance in a structured interview to later struggling. Given the evidence here and in other studies, that early identification of students encountering difficulty is possible, the question becomes that of effective intervention. Conventional responses include periods of suspension, referral for treatment or remediation, and study skills training [23-25]. One feature of the present and Yates’ [1] Nottingham results that is germane, is the group called ‘pre-clinical’ – students who encounter significant difficulties in the early part of the course, but who then progress without further problem. It may be that this group successfully solve their underlying problems – for instance, radically amending their study approach [26] – and closer study of how they achieve this could be beneficial. However, a substantial minority of struggling students could not be identified early in these studies. Reasons for that probably include lack of disclosure by some students and the causes likely include lack of insight [27] and shame or perceived stigma [28-30]. One must also recognise that some of the difficulties students encounter are intractable.

This study has several limitations. It has looked only at a single GEM programme and the numbers involved are small, hence the results may not generalise to other, dissimilar, graduate entry medicine programmes. Data collection was limited to written records contained in the course files and the evaluation of qualitative comments was subjective. No comparison was made with corresponding information from the files of students not encountering difficulty. Although, therefore, it is possible that some characteristics of these struggling students are also present amongst those who do not, it seems unlikely that the markers of difficulty reported here – flags - would occur as more than the occasional one or two flags. However, that is the subject of a planned prospective study that might reveal how students who do not struggle, but encounter similar difficulties, cope successfully with them.

## Conclusions

Problems encountered in a graduate entry medicine course were comparable with those reported in the corresponding undergraduate programme. The toolkit of academic and non-academic markers, or flags, developed for the latter can also be used to identify potential strugglers in graduate-entry medicine at an early stage.

## Abbreviations

GEM: Graduate-entry medicine programme (i.e. leading to initial medical qualification of bachelor of medicine, bachelor of surgery).

## Competing interests

The authors declare that they have no competing interests.

## Authors’ contributions

PG wrote the initial draft of the paper and contributed the admissions data. JY conceived the study, collected and analysed the data, and helped to draft the manuscript. Both authors read and approved the final manuscript.

## Pre-publication history

The pre-publication history for this paper can be accessed here:

http://www.biomedcentral.com/1472-6920/12/124/prepub

## Supplementary Material

Additional file 1 **GEM course structure and selection criteria.** The file contains a diagram of the 4-year graduate-entry medicine programme at University of Nottingham. It also contains a summary of the selection criteria for admission to this programme. Click here for file
