# Supplementary material for: Profiling strugglers in a graduate-entry medicine course at Nottingham: a retrospective case study
Source: BMC Med Educ. 2012 Dec 18;12:124. doi: 10.1186/1472-6920-12-124 (PMC3567936; doi:10.1186/1472-6920-12-124)
Supplement: Additional file 1 — GEM course structure and selection criteria. The file contains a diagram of the 4-year graduate-entry medicine programme at University of Nottingham. It also contains a summary of the selection criteria for admission to this programme. [file 1472-6920-12-124-S1.pdf]

GEM (graduate-entry medicine) Programme, University of Nottingham, UK

Structure of programme (2003-6)

The overall structure comprised 18 months largely pre-clinical phase, followed by a 30 months full-time clinical phase. In the first phase, students take systems-based modules (6 in year 1, 3 in year 2) plus a personal & professional development module in each year. During the full-time clinical phase, students take clinical rotations in medicine & surgery plus modules in therapeutics and a community follow-up project (year 2), attachments in women's health, child health, psychiatry + health care of the elderly, ophthalmology, otorhinolaryngology, and dermatology, plus a student-selected module (year 3), and further rotations in medicine, surgery, general practice, rheumatology + neurology/rehabilitation + orthopaedics, plus a further student-selected module (year 4), concluding with an elective period and a preparation for house officer module.

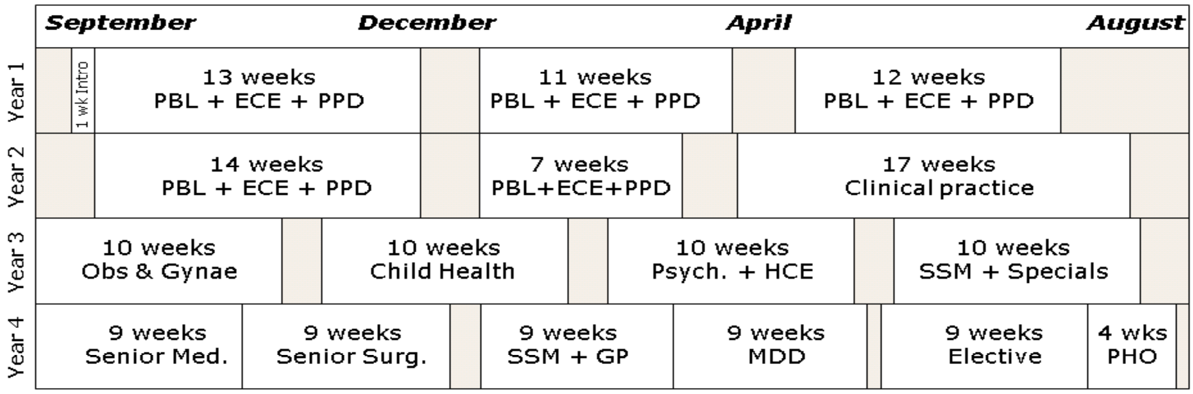

PBL - problem-based learning
ECE - early clinical experience
PPD - personal & professional development
MDD - musculo-skeletal disorders & disability
Obs & gynae - obstetrics & gynaecology
Psych - psychiatry
SSM - special study module (choice of options)
PHO - preparation for house officer

Admissions criteria & process (2003-6)

The admissions process for the Nottingham GEM programme comprised:-

1. Application via UCAS (Universities and Colleges Admissions Service)
2. Eligible with 1st or 2nd (upper or lower) Class Honours degree in any subject
3. Take GAMSAT (Graduate Australian Medical Schools Aptitude Test)
4. GAMSAT cut-off score set (highest overall GAMSAT: either arithmetic mean of Sections I, II & III; or weighted mean of Section I, II, and 2* Section III score) – ranged 59-62 for 2003-6 entry cohorts
5. Attend structured interview assessing personal qualities (e.g. empathy, integrity, insight, communication skills) – interview performance graded A (outstanding) to E (unsuitable)
6. Places offered on basis of a) interview grade, and b) GAMSAT highest mean score within each interview grade.
